# ERK5 Contributes to VEGF Alteration in Diabetic Retinopathy

**DOI:** 10.1155/2010/465824

**Published:** 2010-06-30

**Authors:** Yuexiu Wu, Yufeng Zuo, Rana Chakrabarti, Biao Feng, Shali Chen, Subrata Chakrabarti

**Affiliations:** Department of Pathology, Schulich School of Medicine, University of Western Ontario, London, ON, Canada N6A 5A5

## Abstract

Diabetic retinopathy is one of the most common causes of blindness in North America. Several signaling mechanisms are activated secondary to hyperglycemia in diabetes, leading to activation of vasoactive factors. We investigated a novel pathway, namely extracellular signal regulated kinase 5 (ERK5) mediated signaling, in modulating glucose-induced vascular endothelial growth factor (VEGF) expression. Human microvascular endothelial cells (HMVEC) were exposed to glucose. In parallel, retinal tissues from streptozotocin-induced diabetic rats were examined after 4 months of follow-up. In HMVECs, glucose caused initial activation followed by deactivation of ERK5 and its downstream mediators myocyte enhancing factor 2C (MEF2C) and Kruppel-like factor 2 (KLF2) mRNA expression. ERK5 inactivation further led to augmented VEGF mRNA expression. Furthermore, siRNA mediated ERK5 gene knockdown suppressed MEF2C and KLF2 expression and increased VEGF expression and angiogenesis. On the other hand, constitutively active MEK5, an activator of ERK5, increased ERK5 activation and ERK5 and KLF2 mRNA expression and attenuated basal- and glucose-induced VEGF mRNA expression. In the retina of diabetic rats, depletion of ERK5, KLF2 and upregulation of VEGF mRNA were demonstrated. These results indicated that ERK5 depletion contributes to glucose induced increased VEGF production and angiogenesis. Hence, ERK5 may be a putative therapeutic target to modulate VEGF expression in diabetic retinopathy.

## 1. Introduction

Diabetic retinopathy (DR) is a devastating complication of diabetes, manifesting primarily as vascular structural and functional changes in the retina, eventually leading to vision loss. DR is the most common cause of blindness in North America in the 25–74-years age group [1]. Glucose-induced increased production of vasoactive factors are characteristics of all chronic diabetic complications including DR. Vascular endothelial growth factor (VEGF) is a key vasoactive factor, which is upregulated in the retina in DR. VEGF is an important mediator of increased vascular permeability in early DR and a major contributor of retinal neovascularization in proliferative DR [1–4]. In human, VEGF mRNA expression is increased in the neovascular membranes from diabetic patients obtained by vitrectomy compared to those removed from the nondiabetic individuals [4]. Augmented VEGF protein production was further observed in human retina in diabetes using immunohistochemistry [2, 3]. We and others have demonstrated increased VEGF expression in the retina of streptozotocin-induced diabetic rat model causing increased microvascular permeability [5, 6]. In a murine model of ischemic retinopathy, inhibition of VEGF has also been shown to suppress retinal neovascularization [7]. Furthermore exposure of endothelial cells (ECs) to high glucose causes increased VEGF expression [8, 9]. VEGF mRNA and protein expression are stimulated by long-term high glucose treatment in bovine microvascular retinal ECs [10]. Previous studies in our lab have demonstrated that VEGF interacts with other vasoactive factors such as endothelin-1 (ET-1) in mediating glucose-induced increased permeability in the ECs [5, 8]. These data indicate that VEGF plays an important role in causing increased vascular permeability and angiogenesis in DR [2, 3]. Several glucose induced signaling mechanisms such as protein kinase C (PKC) activation, nonenzymatic glycation and mitogen-activated protein kinase (MAPK) activation are instrumental in causing glucose induced alteration of vasoactive factors in diabetes [11]. 

ERK5, also known as big MAPK1 (BMK1), was identified as a member of the MAPK family in 1995 [12, 13]. ERK5 is a protein of 816 amino acid residues with a large COOH terminal. BMK1 is different from other MAPK as it has a transcriptional activation domain. MAPK/ERK kinase 5 (MEK5) is the specific MAPK kinase for ERK5. ERK5 is highly expressed in the ECs [14]. Studies on ERK5 knockout mice have shown that the ERK5 pathway is critical for endothelial function and for maintaining blood vessel integrity [15]. In addition, ERK5 signaling mediates stress response in the ECs [14, 16]. More recently, studies have shown that ERK5 signaling controls migration and morphology of the ECs [17].

It has been demonstrated that in the nondiabetic conditions, ERK5 have a regulatory role on VEGF expression [18–20]. In mouse, mutant ERK5 gene (homozygous knockout) increases expression of VEGF mRNA and protein [19, 20]. Moreover, ERK5 represses VEGF expression in bovine lung microvascular ECS [18]. Investigations have shown that overexpression of Krupple-like factor 2 (KLF2), a target gene of ERK5, inhibited VEGF receptor promoter activation [21]. KLF2 overexpression also counteracts VEGF-mediated inflammatory responses in the ECs [22]. In addition, microarray studies have revealed that KLF2 overexpression decreases mRNA expression of human VEGF receptor 2 [23, 24]. Hence it is potentially possible that ERK5 through KLF2 may also have a regulatory role on the production of VEGF in DR. However such possibilities have not been investigated. Here, we examined the mechanisms of glucose-induced ERK5-mediated regulation of VEGF in the ECs and in the retina of diabetic rats. We further explored the significance of such changes in DR.

## 2. Materials and Methods

### 2.1. Cell Culture

A dermal-derived human microvascular endothelial cell (HMVEC) was obtained from Lonza, Inc. (Walkersville, MD). HMVECs were grown in endothelial cell basal medium 2 (EBM-2, Lonza, Walkersville, MD) containing human epidermal growth factor (hEGF), 1‰; Hydrocortisone, 0.4‰; gentamycin, 1‰; fetal bovine serum (FBS), 10‰; vascular endothelial growth factor (VEGF), 1‰; human fibroblast growth factor-basic (hFGF-B), 4‰; long R3 insulin-like growth factor (R3-IGF-1), 1‰; Ascorbic Acid, 1‰. In EBM2, the glucose concentration was 5 mmol/l. Cells were grown in 25 cm^2^ tissue culture flasks and maintained in a humidified atmosphere containing 5% CO_2_ at 37°C. Cells at 80% confluence were growth arrested by incubation in serum-free medium overnight prior to incubation with high glucose (25 mmol/l D-glucose) or osmotic control (L-glucose) of the same concentration.

### 2.2. Gain and Loss of Function Studies

For gain of function study, constitutively active human recombinant MEK5 (CAMEK5, 20MOI) adenovirus (Cell Biolabs, San Diego, CA) was used to activate ERK5. Cells were seeded in 6-well plate, cultured overnight and infected with adenovirus for 48 hrs. A nonspecific GFP adenovirus with the same multiplicity of infection (20MOI) was used as a negative control. For loss of function study, ERK5 siRNA (siERK5) was used to knock down ERK5 expression in endothelial cells. Endothelial cells were transfected with ERK5 siRNAs (ON-TARGET*plus* siRNA, 100 nmol/l; Dharmacon Inc. Lafayette, CO) for 48 hrs using siRNA transfection reagent (DharmaFECT 4; Dharmacon Inc. Lafayette, CO) as described before in [25]. A nontargeting siRNA (siGENOME NonTargeting Pool; Dharmacon Inc. Lafayette, CO) with the same concentration of ERK5 siRNA was used as a negative control (control siRNA). siRNA knock down efficiency was determined by real-time RT-PCR.

### 2.3. RNA Isolation and cDNA Synthesis

TRIzol reagent (Invitrogen, Burlington, ON, Canada) was used to isolate RNA as previously described in [26]. RNA was extracted with chloroform followed by centrifugation to separate the sample into aqueous and organic phases. RNA was recovered from the aqueous phase by isopropyl alcohol precipitation and suspended in diethylpyrocarbonate-treated water. Total RNA (2–4 *μ*g) was used for cDNA synthesis with High Capacity cDNA Reverse Transcription Kit (Applied Biosystem, Foster City, CA). The resulting cDNA products were stored at −20°C.

### 2.4. Real-Time RT-PCR

Real-time RT-PCR was performed using the LightCycler (Roche Diagnostics Canada, Laval, PQ, Canada) as previously described in [27]. For a final reaction volume of 20 *μ*l, the following reagents were added: 10 *μ*l SYBR Advantage qPCR Premix (Clontech, Mountain View, CA), 1 *μ*L of each forward and reverse 10 *μ*M primers (Table 1), 7 *μ*L H_2_O, and 1 *μ*L cDNA template. Messenger RNA (mRNA) levels were quantified using the standard curve method. Standard curves were constructed by using serially diluted standard template. The data were normalized to 18S ribosomal RNA or *β*-actin RNA to account for differences in reverse transcription efficiencies and the amount of template in the reaction mixtures.

### 2.5. Western Blot Analysis

Total proteins from endothelial cells were isolated by homogenizing cells in lysis buffer (contains 25 mmol/l Tris*·*HCl, pH 7.5, 150 mmol/l NaCl, 5 mmol/l MgCl_2_, 1% NP-40, 1 mmol/l DTT and 5% glycerol) and protease inhibitor (complete Mini tablet, Roche) and phosphatase inhibitor cocktail 1 and 2 (Sigma-Aldrich, Saint Louis, MO). Protein concentrations were determined by bicinchoninic acid (BCA) protein assay kit (Pierce, Rockford, IL). 30 *μ*g of protein was resolved by 10% sodium dodecyl sulfate-polyacrylamide gel electrophoresis and transferred to a PVDF membrane (BIO-RAD, Hercules, CA). The membrane was then incubated with the rabbit antiphospho-ERK5 antibody (1 : 1000; Cell Signaling Technology, MA). Horseradish peroxidase-conjugated antirabbit antibody (1 : 10000; Upstate Biotechnology, Charlottesville, VA) was used for detection. The signals from the western blots were visualized with an ECL plus chemiluminescence detection kit (Amersham Pharmacia Biotechnology, Buckinghamshire, UK). Blots were stripped with ReBlot Plus Strong Antibody Stripping Solution (Millipore Corporation, Billerica, MA) and reprobed with ERK5 antibody (1 : 1000; Cell Signaling Technology, MA). Blots were then stripped again and reprobed with *β*-actin antibody (1 : 1000, Santa Cruz Biotechnology, CA) as a control for protein loading. Blots were quantified by densitometry using Mocha software (SPSS, Chicago, IL) and the data expressed as a ratio of phosphor-Erk5 to *β*-actin.

### 2.6. In Vitro Angiogenesis Assay

The angiogenic responses to high glucose (25 mmol/l) and ERK5 siRNA transfection were assessed using an in vitro Matrigel analysis. Following appropriate treatment, HMVECs were seeded in 96-well culture plates precoated with ECMatrix (In Vitro Angiogenesis Assay Kit, Milipore, Billerica, MA) at 1 × 10^4^ cells/well. Cells were maintained in serum-free medium at 37°C for 6 hrs. The tube-like structures were visualized by a Leica inverted light microscope. Images were captured with Infinity Capture software Version 3.5.1 at ×10 magnification after 6 hrs incubation. To quantify the image of tube formation, branch points were counted in several random microscopic fields (3–5) per sample and the values averaged. At least 3 different cultures were counted per experimental group. The data were expressed as number of branch points per100× field.

### 2.7. Animal Experiments

Male Sprague-Dawley rats (Charles River) weighing between 200 and 250 g were used. Diabetes was induced by a single intravenous injection of streptozotocin (65 mg/kg, in citrate buffer, pH 5.6). Age-and sex-matched rats were used as controls and given equal volume of citrate buffer [28]. The animals were monitored for glucosuria and ketonuria (Uriscan Gluketo; Yeong Dong, Seoul, South Korea). All diabetic rats were implanted with slow release insulin implants to prevent ketosis (approximately 2 U/day) (LinShin, Scarborough, ON, Canada). They were sacrificed after 4 month of diabetes. We have previously demonstrated that they develop diabetes induced tissue damage in the retina and kidney at this time [29]. The eyes were immediately enucleated, lens and vitreous removed. The retinas of the right eye were gently peeled off, snap-frozen in liquid nitrogen, and stored at −70°C. The left retinas were fixed in 4% paraformaldehyde (PFA), as described before in [5]. All animals were cared for according to the Association for Research in Vision and Ophthalmology*'*s *Guiding Principles in the Care and Use of Animals*. All experiments were approved by the University of Western Ontario Council on Animal Care Committee.

### 2.8. Immunohistochemistry

Formalin fixed retinal tissues were embedded in paraffin, sectioned at 4 *μ*M thickness, and placed on positively charged slides for phosphor-ERK5 (pERK5) immunohistochemical staining. Briefly, the sections were incubated with rabbit anti-pERK5 antibody (Invitrogen, Carlsbad, CA, USA) at 1 : 200 dilutions overnight at 4°C, followed by incubation with labeled polymer-HRP anti-rabbit antibody (Dako North America, Carpinteria, CA, USA) for 30 minutes at room temperature. Visualization was conducted using diaminobenzidine (DAB, Dako North America, Carpinteria, CA, USA) as HRP substrate. Slides were counterstained with Hematoxylin. Staining with nonimmune rabbit serum instead of primary antibodies was used as negative controls. Images were recorded by an Olympus BX51 microscope (Olympus Canada Inc, ON, Canada) with Northern Eclipse software (Empix Inc, ON, Canada).

### 2.9. Statistical Analysis

Data were presented as the mean ± standard error. Statistical significance of difference between groups was tested using Student's *t*-test or if there were more than two groups, using one way analysis of variance (ANOVA) followed by posthoc analysis. A *P* value of  .05 or less was considered to be significant. All calculations were performed using SPSS version 15.0.

## 3. Results

### 3.1. Glucose Caused ERK5 Alteration and VEGF Upregulation

**Figure 1:**
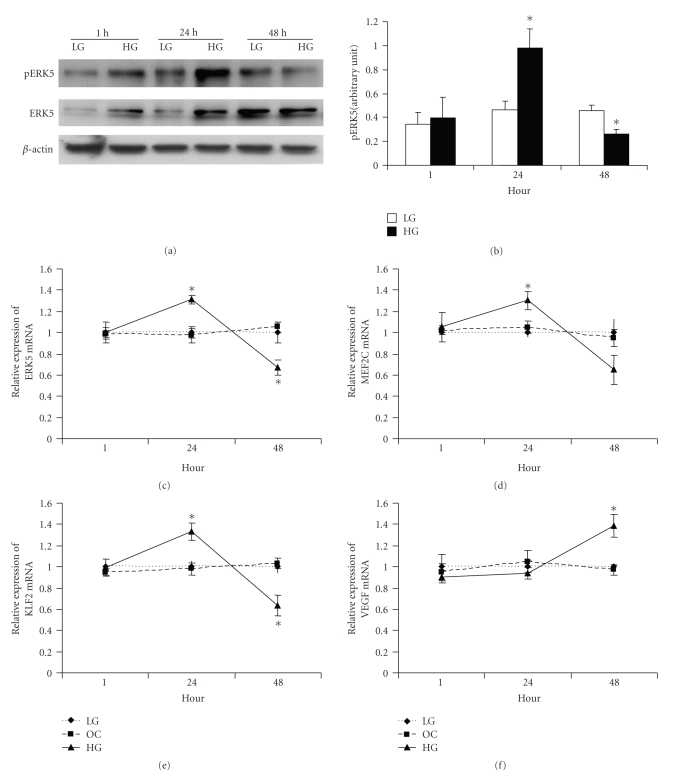
(a) Representative Western Blot showing increased pERK5 and total ERK5 after 24 hrs HG treatment which subsequently decreased after 48 hrs. (b): Densitometric quantification of pERK5 expression. (c–f): Real-time PCR showed that following incubation in HG, ERK5, MEF2C and KLF2 mRNA expression were increased after 24 hrs and then were decreased after 48 hrs. On the other hand, VEGF mRNA expression did not increase until after 48 hrs of HG treatment. LG : 5 mmol/l D-glucose; OC: osmotic control, 25 mmol/l L-glucose; HG: 25 mmol/l D-glucose. [Data in “B” are expressed as a ratio to *β*-actin. mRNAs are expressed as a ratio to 18S, normalized to controls, * significantly different from LG, *n* = 3 − 6/group.]

We initially established whether in the ECs, glucose causes any alteration of ERK5 signaling. No change in the mRNA expression of ERK5 and its downstream molecules MEF2C, KLF2, or VEGF were seen after exposure of the cells to 25 mmol/l glucose (HG) for 1 hour compared to 5 mmol/l glucose (LG) (Figures 1(c)–1(f)). L-glucose was used as an osmotic control (OC). Following 24 hrs of glucose exposure, ERK5 signaling was significantly activated as evidenced by increased phosphorylation (Figures 1(a) and 1(b)) and mRNA expression of ERK5 (Figure 1(c)) and augmented mRNA expression of MEF2C (Figure 1(d)) and KLF2 (Figure 1(e)), which are downstream mediators of ERK5 signaling. Interestingly, at this time point there were no increases of VEGF mRNA expression (Figure 1(f)). However, with increased duration of high-glucose treatment (48 hrs), ERK5 phosphoryation and mRNA expression (Figures 1(a)–1(c)) as well as MEF2C and KLF2 mRNA expression (Figures 1(d) and 1(e)) were decreased, while VEGF mRNA expression was increased (Figure 1(f)). Similar results were also observed after 72 hrs of HG treatment (data not shown). Since expression of total ERK5 was also changed after glucose treatment, western blot of pERK5 was normalized to *β*-actin. To further delineate the mechanistic role and significance of glucose-induced ERK5 activation, especially with its regulatory effects on VEGF expression, gain and loss of function studies were performed.

### 3.2. ERK5 Downregulation Led to Increased VEGF Expression

**Figure 2:**
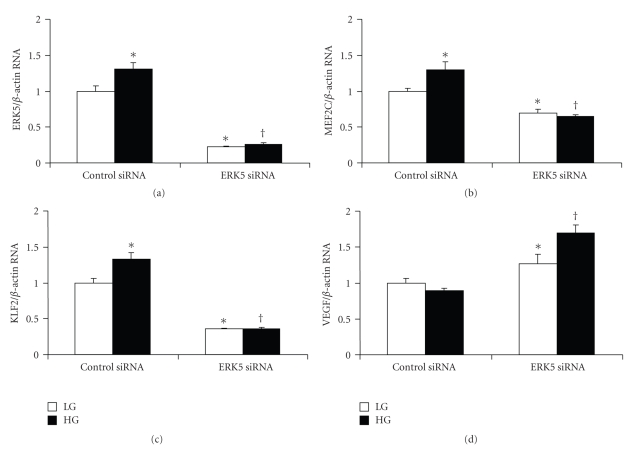
ERK5 siRNA transfection reduced basal (5 mmol/l, LG) and glucose induced (25 mmol/l, HG) mRNA expression of ERK5 (a), MEF2C (b), KLF2 (c) in endothelial cells after 24 hrs of HG treatment. On the other hand, such transfection augmented VEGF mRNA expression (d). [Data are expressed as a ratio to *β*-actin, normalized to controls, *: significantly different from control siRNA in LG, **: significant difference from control siRNA in HG, *n* = 6/group].

Our initial investigation indicated that VEGF mRNA expression was inversely related with ERK5 activation following high-glucose treatment. To further explore the interaction of ERK5 signaling and VEGF expression, loss of function study was performed in the HMVECs using ERK5 siRNA. ERK5 siRNA was transfected to endothelial cells to knock down ERK5 gene. Real-time PCR analyses demonstrated that such transfection led to >70% reduction of ERK5 mRNA expression (Figure 2(a)). ERK5's downstream substrates, MEF2C and KLF2, were also significantly reduced (Figures 2(b) and 2(c)). As expected, VEGF mRNA expression was increased following ERK5 siRNA transfection measured by real-time PCR (Figure 2(d)). To further study the effect of ERK5 knockdown on glucose induced VEGF upregulation, HMVECs were transfected with ERK5 siRNA and then treated with high glucose for short period (24 hrs). Results showed that ERK5 siRNA transfection abolished high-glucose-induced increase of ERK5, MEF2C, KLF2 mRNA (Figures 2(a)–2(c)), whereas promoted upregulation of VEGF mRNA (Figure 2(d)).

### 3.3. Functional Significance of Glucose-Induced–ERK5 Mediated VEGF Upregulation

**Figure 3:**
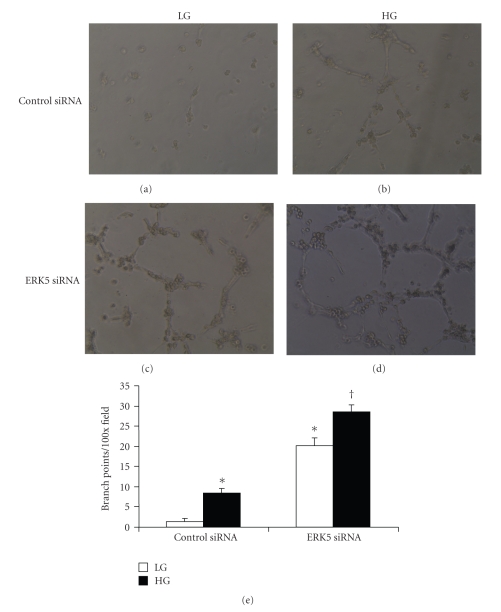
ERK5 siRNA enhanced tube formation in ECs under normal and high glucose conditions. (a–d): Representative phase-contrast photographs of in vitro angiogenesis assay showing tube formation in ECs. Such tube formations were pronounced following ERK5 siRNA transfection ((c) and (d)), compared with control siRNA transfection ((a) and (b)), both in 5 mmol/l (LG) or 25 mmol/l (HG) glucose. Original magnification at 100x. (e): Quantification of tube formation by counting branch points of tube-like structures confirmed stimulatory effect of ERK5 siRNA on tube formation. [Data are expressed as number of branch points per 100× field, * significantly different from control siRNA in LG, †: significant difference from control siRNA in HG, *n* = 3/group].

We further expanded the investigations to examine functional significance of glucose-induced ERK5 mediated VEGF expression. As glucose-induced VEGF upregulation plays an important role in neovascularization in proliferative DR, we examined whether alteration of ERK5 and subsequent change in VEGF has any effects on endothelial tube formation using and an *in vitro* angiogenesis assay. High-glucose treatment (48 hrs) stimulated branching and tube formation in endothelial cells transfected with control siRNA (Figures 3(a) and 3(b)), which was similar as our finding in endothelial cells without transfection (data not shown). Glucose induced tube formation paralleled increased VEGF mRNA expression measured by real-time PCR (Figure 1(e)). Endothelial cells transfected with ERK5 siRNA rapidly formed capillary-like tube structures (Figure 3(c)). High-glucose treatment further augmented the number and size of tube-like structure formation (Figure 3(d)). Quantification of tube formation is shown in Figure 3(e). Such increase in tube formation was associated with pronounced VEGF mRNA expression (Figure 2(d)). These results suggest that decreased ERK5 stimulates angiogenesis by increasing VEGF expression.

### 3.4. ERK5 Upregulation Inhibited VEGF Expression in Endothelial Cells

 We then investigated whether ERK5 upregulation can protect endothelial cells in pathological conditions mediated by glucose. As glucose-induced tissue damage is mediated by vasoactive factors such as VEGF, we proceeded to examine whether glucose induced upregulation of VEGF mRNA can be prevented by constitutively active MEK5 (CAMEK5).

**Figure 4:**
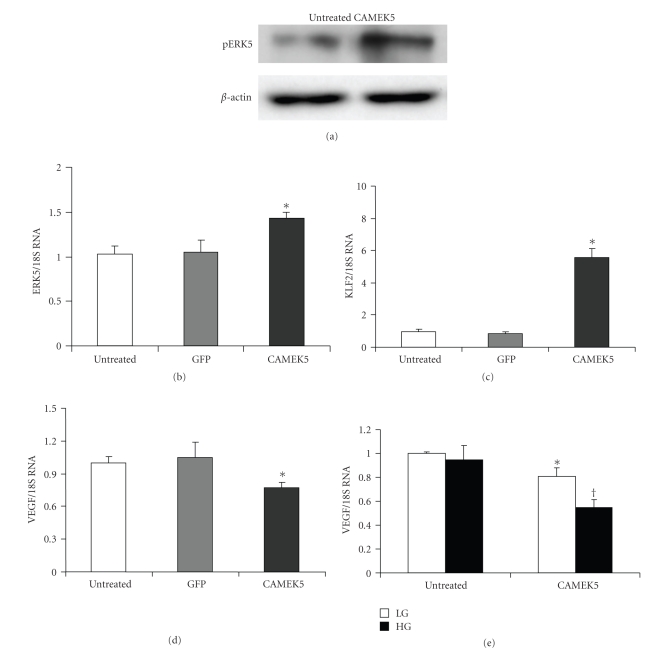
In ECs, constitutionally active MEK5 (CAMEK5) caused increased ERK5 phosphorylation as indicated by (a) Western blot using phospho ERK5 antibody. CAMEK5 also caused mRNA upregulation of ERK5 (b), KLF2 (c) and downregulation of VEGF (d). Twenty-four hours exposure of 25 mmol/l of glucose (HG) significantly decreased VEGF mRNA expression after CAMEK5 infection (e). [mRNAs are expressed as a ratio to 18S, normalized to control, *n* = 5/group, *: significantly different from untreated or GFP controls, †: significantly different from other groups].

MEK5 is a specific MAPK kinase for ERK5 [13, 30, 31]. Hence, use of CAMEK5 adenovirus to upregulate ERK5 signaling is a rational approach. CAMEK5 not only activated ERK5 phosphorylation, but also augmented ERK5 transcription (Figures 4(a) and 4(b)). Western Blot confirmed increased pERK5 after CAMEK5 infection in HMVECs (Figure 4(a)). Real-time PCR showed that ERK5 mRNA level after CAMEK5 infection was significantly higher than that of GFP control and untreated control (Figure 4(b)). ERK5 activation caused upregulation of KLF2 mRNA expression (Figure 4(c)) and downregulation of VEGF mRNA expression. (Figure 4(d)). 

To study the effect of CAMEK5 on glucose-induced VEGF expression, HMVECs was infected with CAMEK5 and then treated with high glucose for 24 hrs. VEGF mRNA was slightly decreased by CAMEK5-induced activation of ERK5 in LG groups, while constitutive activation of ERK5 by CAMEK5 infection led to a significant decrease of VEGF after HG treatment (Figure 4(e)).

### 3.5. Reduced ERK5 Activation Is Associated with Increased VEGF mRNA Expression in Retinas of Diabetic Rats

**Figure 5:**
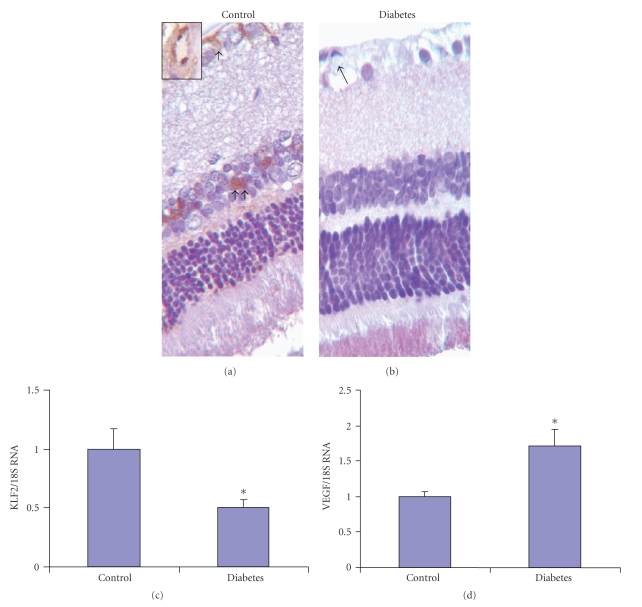
Immunohistochemical staining of pERK5 in the retina showing reduced pERK5 protein expression in the retina of diabetic rats (b) compared to the control animals (a). Positive pERK5 staining was localized in the ganglion cell and inner nuclear layers (arrows). Inset is an enlarged view of a microvessel showing positive pERK5 staining in the endothelial cells. Real-time PCR analysis showed reduced KLF2 (c) and augmented VEGF (d) mRNA expression in the retina of diabetic rats compared to the controls. [Original magnification at 100x. mRNAs are expressed as a ratio to 18S, normalized to control, *n* = 5/group, * significantly different from controls.]

From the perspective of DR, it is important to examine whether the alterations demonstrated in the endothelial cells are indeed important in a clinically relevant model of diabetic retinal microangiopathy. Hence, we investigated retinas from a well-established model of diabetic retinal microangiopathy. Phospho-ERK5 immunohistochemical staining was performed in the retinal tissues of STZ-induced diabetic rats after 4 month of followup. Diabetic animals showed increased blood glucose levels, reduced body weight gain, glucosuria, and occasional ketonuria (data not shown). Positive pERK5 staining was localized in the ganglion cell layer and in the inner nuclear layer. Microscopic examination further revealed that such positivity was in the microvasculature as well as in other cells. The number of positive cells and the intensity of staining were reduced in the retina of diabetic animals compared with that of controls (Figures 5(a) and 5(b)). We also examined the mRNA expression of ERK5, KLF2, and VEGF in retinal tissues of 4-month diabetic and control rats. Real-time PCR showed that ERK5 mRNA expression in diabetic group was too low to be detected (data not shown). In parallel, mRNA expression of KLF2, a downstream molecule of ERK5 signaling, in diabetic group was markedly lower than that of control group (Figure 5(c)). In keeping with our previous data from our lab and others, real-time PCR analyses demonstrated a significant increase of VEGF mRNA expression in the retina of diabetic rats (Figure 5(d)).

## 4. Discussion

Here we have demonstrated a novel mechanism of glucose mediated VEGF gene upregulation in diabetes. We have shown that in the ECs glucose causes transient activation of ERK5 followed by deactivation. Reduced activity of ERK5 was associated with upregulation of VEGF mRNA and angiogenesis. We have confirmed such negative regulation of ERK5 on VEGF using ERK5 silencing and overexpression. Furthermore, we have found similar alteration of ERK5 and VEGF in the retina of diabetic animals.

A substantial body of evidence indicates that VEGF is a major angiogenic factor involved in DR [32, 33]. MAPK signaling pathways play essential roles in modulating expression of VEGF [34]. Constitutive activation of ERK1/2 elevated expression of VEGF mRNA [35]. Overexpression of p38 and JNK activation increased halflife of VEGF mRNA [36]. In addition, knockout animal study showed that JNK regulated VEGF expression at the transcriptional level in hypoxia induced retinal VEGF production [37]. A recent publication revealed that Wnt signaling is activated in DR and upregulates VEGF expression [38]. On the other hand ERK5, as demonstrated in this paper, is the only protective signaling that is activated by high glucose. In this study, we examined the effect of ERK5 on high-glucose-induced VEGF expression and demonstrated a novel pathway that potentially contributes to VEGF expression and subsequent angiogenesis in DR. 

ERK5 is different from other MAPKs because of its unique C-terminal, which contains transcriptional activation domain. Transcriptional activation can enhance the effect of ERK5 signaling [30]. Hence, we examined ERK5 transcription in our study which paralleled its downstream effects. Expression of this transcription domain is sufficient to drive MEF2 and regulate MEF2-dependent gene expression [39]. The importance of transcriptional activation ERK5 have also been previously demonstrated by its effects on inhibition of ERK5 SUMOylation and prevention of diabetes-mediated left ventricular dysfunction [40]. However, ERK5 also activates signaling using traditional phosphorylation [41]. Studies have shown that the activated kinase activity of ERK5 undergoes autophosphorylation on its most C-terminal region, which is required for the C-terminal-half to enhance the ERK5 activity [30]. In this study we have demonstrated both attenuated ERK5 transcription and phosphorylation in high-glucose treated endothelial cells. Our data indicate that both mechanisms may be operating in glucose-induced VEGF upregulation.

It has been previously reported that MEF2-KLF2 counteracted VEGF-mediated inflammatory responses in endothelial cells [22]. In addition, KLF2, a downstream molecule of ERK5, is a transcriptional regulator of angiogenesis, and overexpression of KLF2 counteracts VEGF-mediated angiogenesis due to a potent inhibition of VEGFR2 expression and promoter activity [21]. Our study found that reduced ERK5, MEF2C, and KLF2 expression were along with increased VEGF level after 48 hrs glucose exposure (Figure 1). On the other hand, after 24 hrs, although ERK5 was activated, we failed to observe VEGF upregulation. Exact reason for such findings is not clear. Possible explanation may include other regulatory factors and cell specific factors, which need further characterization. Nevertheless, ERK5 siRNA transfection significantly reduced MEF2C and KLF2 mRNA expression (Figure 4), suggesting that ERK5 negatively regulates VEGF through MEF2C and KLF2 upon high glucose treatment. Under hypoxic conditions, ERK5 inhibits VEGF via hypoxia inducible factor 1*α* (HIF1*α*) in endothelial cells [18]. In addition, it has been shown that KLF2 inhibits HIF1a and hypoxia-mediated angiogenesis [42]. High glucose induced a state of pseudo-hypoxia in diabetic complications [43, 44]. It is therefore possible that depletion of ERK5/KLF2 signaling may promote high glucose-induced angiogenesis via HIF1a. However, such notion has to be further established by specific experiments.

In keeping with our finding it has been demonstrated that ERK5 activation is induced transiently by high glucose in endothelial cells, which ultimately decreased after long-term treatment [45]. As following long-term glucose exposure in ECs and in the retina of chronically diabetic animals, similar pattern of ERK5 and VEGF were seen, it is possible that in long-term diabetes inhibitory effects of ERK5 is lost, leading to VEGF upregulation. In this study we have seen VEGF upregulation after 48 hrs. This is in keeping with studies in endothelial cells from other sources [9, 46]. Some studies have demonstrated VEGF upregulation following a short period of glucose exposure. Various sources of cells and culture conditions may in part be responsible for such discrepancy. In addition simultaneously other mechanisms may also be responsible for glucose induced VEGF upregulation. Both PKC and ERK1/2 activation have been demonstrated to regulate VEGF in glomerular podocytes [47]. In this study we observed that after 24 hrs glucose treatment, although there was significant activation of ERK5, there were no significant downregulation of VEGF in this system. This suggests that other mechanisms mentioned above may also modulate glucose-induced VEGF, further investigation is required to delineate the relationship of ERK5 and other signaling pathways. 

It is well established that oxidant and shear stress can regulate ERK5 alteration. Exact mechanism of glucose induced ERK5 alteration is still not known, however it is possible that glucose-induced oxidative stress is a key player in ERK5 change. Growth factors such as epidermal growth factor (EGF) and nerve growth factor (NGF) activate ERK5 [48, 49]. Hence several additional mechanisms may potentially regulate glucose induced ERK5 alteration. We, however, understand some of the limitations of this study as the in vitro experiments were performed in the microvascular cells of nonretinal origin. We tried to address some of these problems with simultaneous experiments at levels of complexities, that is, retinal tissues. However, additional future studies are needed in various cell types to further characterize these changes.

In summary, this is the first study to show that ERK5 may potentially regulate VEGF upon high-glucose treatment in the ECs and in the retina of diabetic rats. Although this study was done in the context of diabetic retinopathy, it is possible that such process is of importance in other diabetic complications involving VEGF signaling. ERK5 may also provide an attractive target for drug development in DR and other diabetic vascular complications.

## Figures and Tables

**Table 1:** Oligonucleotide sequences for real-time PCR.

Gene	Sequence 5′ → 3′
ERK5 (human)	CTGGCTGTCCAGATGTGAA
	ATGGCACCATCTTTCTTTGG
MEF2C (human)	TACAACGAGCCGCATGAGAG
	CCTGTGTTACCTGCACTTGG
KLF2 (human)	GCACGCACACAGGTGAGAAG
	ACCAGTCACAGTTTGGGAGGG
VEGF (human)	GGCCTCCGAAACCATGAACTTTCTGCT
	GCATGCCCTCCTGCCCGGCTCACCGC
VEGF (rat)	CTGCTGTCTTGGGTGCATTGG
	CACCGCCTTGGCTTGTCACAT
*β*-actin (human and rat)	CCTCTATGCCAACACAGTGC
	CATCGTACTCCTGCTTGCTG
18S (human and rat)	GTAACCCGTTGAACCCCATT
	CCATCCAACGGTAGTAGCG
